# Structural and functional alterations of the hippocampal subfields in T2DM with mild cognitive impairment and insulin resistance: A prospective study

**DOI:** 10.1111/1753-0407.70029

**Published:** 2024-11-13

**Authors:** Chen Yang, Huiyan Zhang, Zihan Ma, Yanjun Fan, Yanan Xu, Jian Tan, Jing Tian, Jiancang Cao, Wenwen Zhang, Gang Huang, Lianping Zhao

**Affiliations:** ^1^ The First Clinical Medical College of Gansu University of Chinese Medicine (Gansu Provincial Hospital) Gansu University of Chinese Medicine Lanzhou China; ^2^ School of Clinical Medicine Ningxia Medical University Yinchuan China; ^3^ Department of Radiology Gansu Provincial Hospital Lanzhou China

**Keywords:** hippocampus, insulin resistance, magnetic resonance imaging, mild cognitive impairment, type 2 diabetes mellitus

## Abstract

**Background:**

Type 2 diabetes mellitus (T2DM) is characterized by insulin resistance (IR) and is often accompanied by mild cognitive impairment (MCI). The detrimental effects of T2DM and IR on the hippocampus have been extensively demonstrated. Few studies have examined the effects of IR on structure and function of hippocampal subfields in T2DM‐MCI patients.

**Method:**

A total of 104 T2DM patients were recruited in this prospective study and divided into four groups (T2DM‐MCI‐higherIR, *n* = 17; T2DM‐MCI‐lowerIR, *n* = 32; T2DM‐nonMCI‐higherIR, *n* = 19; T2DM‐nonMCI‐lowerIR, *n* = 36). Structure and function MRI data were captured. Clinical variables and neuropsychological scores were determined for all participants. Hippocampal subfield volume and functional connectivity were compared among four groups. Partial correlation analysis was performed between imaging indicators, clinical variables, and neuropsychological scores in all patients.

**Results:**

T2DM‐MCI‐higher IR group had the smallest volumes of bilateral hippocampal tail, right subiculum‐body, right GC‐ML‐DG‐body, and right CA4‐body. IR in right hippocampal tail, right subiculum‐body, and right GC‐ML‐DG‐body exerted main effect. Furthermore, elevated functional connectivity was found between right subiculum‐body and bilateral dorsolateral prefrontal cortex and right anterior cingulate–medial prefrontal cortex. Hippocampal subfield volume positively correlates with total cholesterol and triglycerides and negatively correlates with fasting insulin.

**Conclusion:**

The present study found that T2DM‐MCI patients have structural and functional alterations in hippocampal subfields, and IR is a negative factor influencing the alteration of hippocampal subfields volume. These findings support the importance of IR in T2DM‐MCI patients and might be potential neuroimaging biomarkers of cerebral impairment in T2DM‐MCI patients.

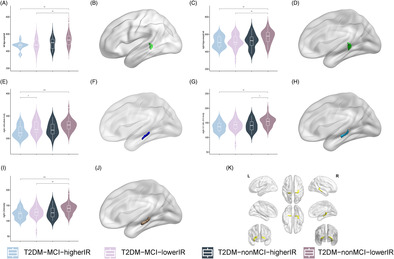

## Graphical abstract

**Highlights**

- T2DM‐MCI with higher insulin resistance has more severe hippocampal volume atrophy.
- Hippocampal‐prefrontal circuitry dysfunction is observed in T2DM patients.
- Insulin resistance plays a crucial role in T2DM patients with cognitive impairment.

## 1 INTRODUCTION

Type 2 diabetes mellitus (T2DM) is a metabolic disorder characterized by hyperglycemia and hyperinsulinemia, and insulin resistance (IR) is a core characteristic of T2DM. In the last few years, the incidence of T2DM has increased worldwide due to improved living standards, changes in lifestyle, and the increasing aging population. A study estimated there to be a total of 642 million diabetics worldwide by 2040.^1^ Previous studies have focused on diabetic microvascular damage, such as diabetic nephropathy, retinopathy, and peripheral neuropathy.^2^, ^3^, ^4^ Recently, diabetes‐related brain impairment has received widespread attention.^5^, ^6^ Mild cognitive impairment (MCI) is characterized by loss or decline of memory and language deterioration.^7^ A study found that T2DM‐MCI patients were more likely to develop Alzheimer's disease (AD) than did community elderly.^8^ Evidence from literature has depicted that brain IR plays an important role in the pathogenic mechanism of AD.^9^, ^10^ T2DM‐induced IR affects regular metabolic processes by disrupting the integrity of the blood–brain barrier and decreasing insulin sensitivity.^11^ Insulin in the central nervous system has been proven to regulate neurosynaptic growth and modulate synaptic plasticity, and the decline in insulin sensitivity due to IR might seriously affect normal metabolic processes in the brain.^12^, ^13^ Intranasal injection of insulin and insulin sensitizers has been shown to improve cognitive function in Streptozotocin rats.^14^, ^15^ In addition, the deposition of amyloid β (Aβ) in neuritic plaques and accumulation of neurofibrillary tangles from tau protein are regarded as two major pathophysiological hallmarks of AD.^16^ This alteration has recently been detected in brain tissue of T2DM, especially in the hippocampus, which further suggests the potential overlap in the pathogenesis of T2DM and AD.^17^, ^18^ However, the role of IR in the cognitive impairment progress in T2DM has not been fully elucidated.

The hippocampus is one of the brain structures more crucially involved in cognitive functions, which is critical for forming long‐term, episodic memories and spatial cognition.^19^, ^20^ Recent experiments demonstrated the hippocampus to be significantly impaired in neurodegenerative diseases such as AD and Parkinson's disease.^21^, ^22^ However, it is a challenge to investigate the hippocampus alteration in vivo. Magnetic resonance imaging as a noninvasive and safe screening method without ionizing radiation has been widely used for neurological diseases even the structural and functional changes of the hippocampus.^23^, ^24^, ^25^ T2DM caused mitochondrial dysfunction in the hippocampus, which impacted the cognitive function in in vivo animal studies.^26^ A voxel‐based morphometry study have revealed reduced hippocampal gray matter volume in patients with T2DM.^27^ Previous studies have mostly focused on the overall structural or functional alterations of the hippocampus, while fewer studies have been conducted on the hippocampal subfields in T2DM patients. However, it is well‐established that the hippocampal subfields exhibit structural or functional heterogeneity.^28^ With the continuous improvement of data processing methods, fine segmentation of hippocampal structure was established for further investigating the changes of hippocampal subfields based on structural MRI.^29^, ^30^ A previous study concerning hippocampal subfields in T2DM‐MCI patients demonstrated a significant reduction in CA1 volume.^31^ Interestingly, studies on rodent models and human post‐mortem brain samples have shown that insulin receptor expression is higher in the hippocampus; consequently, the hippocampus may be more vulnerable to IR‐related cerebral impairment.^32^, ^33^ However, there was no study that investigated volumetric or functional changes in hippocampal subfields in T2DM patients based on the level of IR as far as we know.

The present study focuses on the role of brain IR in structural or functional alterations in hippocampal subfields in T2DM patients with or without MCI using fine segmentation of hippocampal subfields and surface‐based functional connectivity (SBFC) analysis.^34^, ^35^ We hypothesized there was more severe structural or functional impairment of specific hippocampal subfields in T2DM‐MCI patients with IR, and these alterations may correlate with clinical variables and neuropsychological test scores.

## 2 SUBJECTS AND METHODS

### 2.1 Subjects

In this study, T2DM patients from the Department of Endocrinology of Gansu Provincial Hospital, China, were recruited between November 2017 and January 2023. All participants were diagnosed with T2DM based on World Health Organization 1999 diagnostic criteria (fasting blood glucose [FBG] levels ≥7.0 mmol/L and postprandial 2‐h blood glucose levels ≥11.1 mmol/L). A total of 168 patients with T2DM were included in this study.

The inclusion criteria were as follows: (1) age 18–65 years; (2) Han Chinese; (3) right‐handed; (4) with at least 6 years of education; (5) not taking psychotic medications for at least 2 months or transcranial magnetic stimulation for at least 3 months. The exclusion criteria were as follows: (1) organic central nervous system lesions; (2) neurodegenerative changes such as AD and PD; (3) psychiatric disorders such as mood disorders and personality disorders; (4) craniocerebral trauma or surgery history; (5) alcohol dependence and drug abuse; (6) any MRI contraindications; (7) pregnant women, lactating mothers, and those taking oral contraceptives. This study received ethics approval from the Medical Ethics Committee of Gansu Provincial Hospital (2017‐188, 2023‐291). In addition, signed informed consent was obtained after it was explained to all participants.

Participants were divided into four groups based on the scales of Montreal Cognitive Assessment (MoCA, Beijing vision) and homeostasis model assessment IR (HOMA2‐IR), calculated using the HOMA2 calculator software (https://www.dtu.Ox.ac.uk/homacalculator). Groups were divided as follows: T2DM‐MCI‐higherIR group: MoCA < 26, HOMA2‐IR ≧ 1.4; T2DM‐MCI‐lowerIR group: MoCA < 26, HOMA2‐IR < 1.4; T2DM‐nonMCI‐higherIR group: MoCA ≧ 26, HOMA2‐IR ≧ 1.4; T2DM‐nonMCI‐lowerIR group: MoCA ≧ 26, HOMA2‐IR < 1.4.^36^, ^37^, ^38^, ^39^

### 2.2 Demographic data, clinical characteristics, and neuropsychological test

The general characteristics, including age, sex, body mass index (BMI), years of education, disease duration, history of hypoglycemic agents used in the past year, systolic blood pressure (SBP), and diastolic blood pressure (DBP), were recorded. Venous blood from the patients was drawn at 8:00 a.m. (more than 10 h of fasting) to establish FBG, hemoglobin A1c (HbA1c), fasting insulin (FINS), total cholesterol (TC), triglyceride (TG), high‐density lipoprotein (HDL), low‐density lipoprotein (LDL), and C‐reactive protein (CRP). The HOMA2‐IR index was calculated using the HOMA2 calculator software.

Comprehensive neuropsychological tests were administered to all subjects. The Mini‐Mental State Examination (MMSE) was applied to assess possible dementia, whereas the MoCA, which has high sensitivity and specificity for MCI, was developed as a brief screening instrument to assess global cognitive performance. In addition, the 24‐item Hamilton Depression Scale (HAMD‐24) and Hamilton Anxiety Scale (HAMA) were used to evaluate the degree of depressive and anxiety symptoms. The neuropsychological evaluation for each subject was completed by professionally trained investigators on the same day of the MRI scans.

### 2.3 MRI data collection

The brain MRI data were collected using a 3.0‐T MRI scanner (MAGNETOM Skyra, Siemens Healthcare, Erlangen, Germany) with 32‐channel phased array head coil. Participants were asked to keep their head still to minimize the influence of head motion. They were also required to keep their eyes closed and remain awake. First, routine cranial MRI sequence (T1WI, T2WI, T2‐FLAIR) was performed to rule out severe hyperintense white matter lesions (above Fazekas II) and organic central nervous system lesions. Then, structure and functional MRI data were collected.

High‐resolution 3D structural T1‐weighted (3D‐T1WI) images were acquired using magnetization‐prepared rapid gradient echo (MPRAGE) sequence with the following parameters: repetition time (TR) = 2530 ms; echo time (TE) = 2.35 ms; flip angle (FA) = 7°; number of slices = 192; slice thickness = 1 mm; field of view (FOV) = 256 × 256 mm; and matrix = 256 × 256; resulting in an isotropic voxel size of 1 × 1 × 1 mm; total scan time = 5 min 23 s. Rest‐state functional magnetic resonance imaging (rs‐fMRI) data were acquired using the blood oxygenation level–dependent sequence based on the gradient‐echo echo planar imaging: TR = 2000 ms; TE = 30 ms; FA = 90^°^; FOV = 224 × 224 mm; matrix = 64 × 64; slice thickness = 3.5 mm; gap = 0.7 mm; number of slices = 33; time point = 420^40^, ^41^; total scan time = 14 min 8 s. Only those confirmed not to have fallen asleep were included in the study.

### 2.4 MRI data processing

#### 2.4.1 Structural MRI data preprocessing and hippocampal subfields volume calculating

**FIGURE 1:**
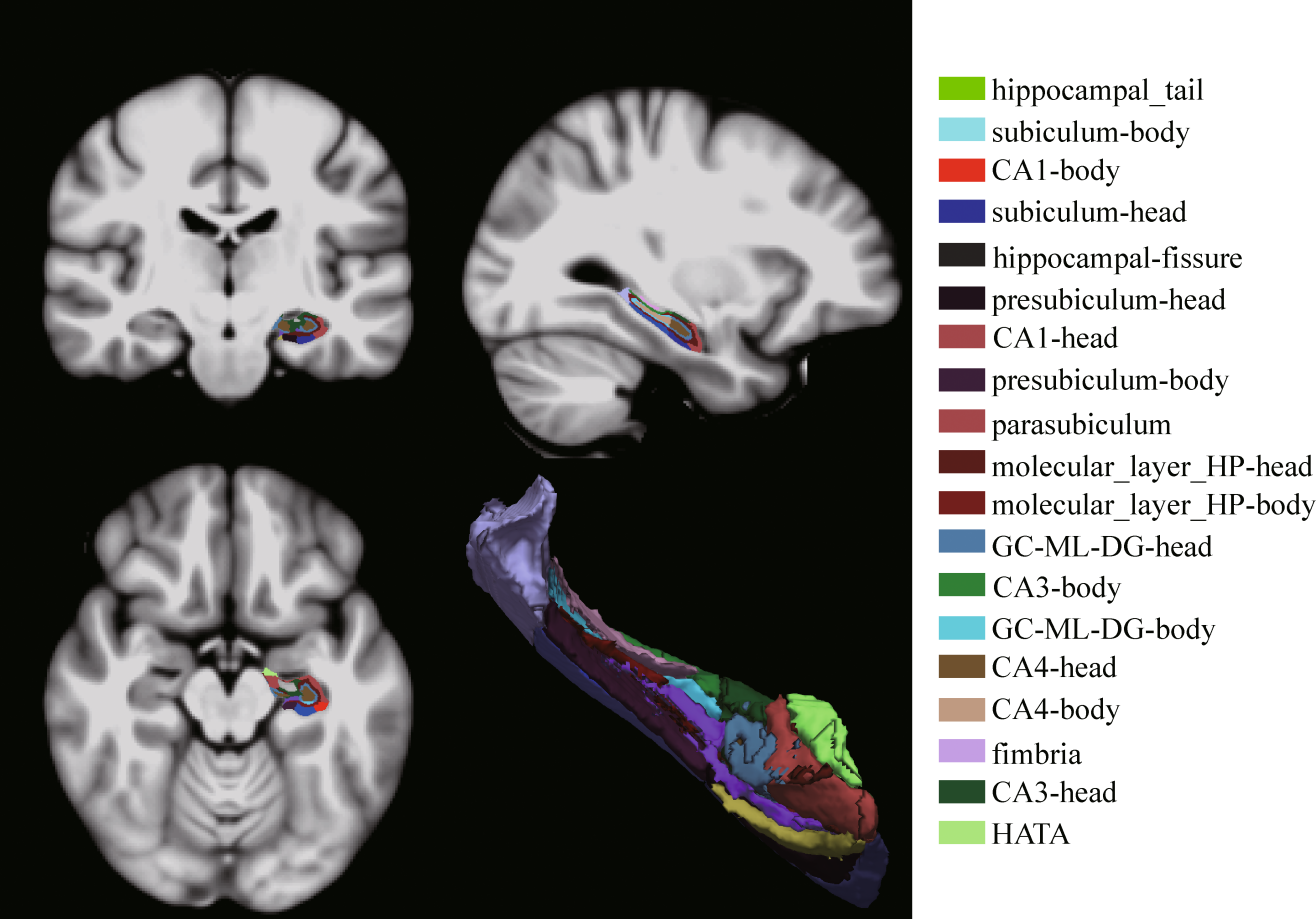
Anatomical location of the hippocampus and its subfields presented in the MNI standard brain; CA, cornu ammonis; GC‐ML‐DG, granule cell layer of dentate gyrus; HATA, hippocampus–amygdala transition area.

3D‐T1WI images were processed‐sectionally in the FreeSurfer v7.2.0 (https://surfer.nmr.mgh.harvard.edu) recon‐all pipeline for automated cortical and subcortical parcellations and tissue segmentation as preprocessing. An automated pipeline for hippocampal subfields segmentation is included in FreeSurfer. Compared to the previous version, the most notable improvement is that it subdivides the hippocampal substructures into head and body. It divided each hippocampal into 19 subfields. These results were used to classify the hippocampal tail, subiculum‐body, CA1‐body, subiculum‐head, hippocampal fissure, presubiculum‐head, CA1‐head, presubiculum‐body, parasubiculum, molecular‐layer‐HP‐head, molecular‐layer‐HP‐body, granule cell layer of dentate gyrus (GC‐ML‐DG)‐body, GC‐ML‐DG‐head, CA3‐body, CA4‐head, CA4‐body, fimbria, CA3‐head, and hippocampus–amygdala transition area (HATA). The volume of hippocampal subfields and estimate total intracranial volume (eTIV) was extracted for subsequent statistical analysis. See Figure 1 for hippocampal subfield segmentation from Montreal Neurological Institute (MNI) standard brain. Enhancing NeuroImaging Genetics through Meta‐Analysis (ENIGMA) studies were followed for quality control of hippocampal segmentation.^42^

#### 2.4.2 Functional MRI data preprocessing and functional data calculation

The anatomical and rs‐fMRI data were preprocessed using the DPABISurf V1.7, which is based on the Statistical Parametric Mapping software (SPM12, http://www.fil.ion.ucl.ac.uk/spm12) and on the Matrix Laboratory platform (MATLAB R2018b, https://www.mathworks.com/), a software used for functional MRI data preprocessing (Supplemental Information 1).

#### 2.4.3 SBFC calculation

Hippocampal subfields that exhibited significant group differences were selected as mask to further calculate the SBFC map. First, the hippocampal subfields with significant group differences were extracted by FreeSurfer as masks from MNI standard space. Then, we calculated Pearson's correlation coefficient between the mean time course of each mask and the time series of each of the remaining voxels throughout the whole brain to generate the SBFC map. Next, the functional connectivity (FC) maps were converted to *z*‐values using Fisher's *r‐z* transformation for standardization for further statistical analysis.

### 2.5 Statistical analysis

The SPSS software (version 20.0, Inc. Chicago, IL, USA) was used for statistical analysis, including demographic, clinical, and neuropsychological test scores. One‐way analysis of variance (ANOVA) and Bonferroni post hoc tests were used for continuous variables with normal distributions and homogeneous variances. Whether the data did not conform to normal distribution or homogeneity of variance, the data were analyzed using the rank‐sum test. Differences in sex distribution were assessed using the chi‐square test. The comparison of the differences in the usage rates of different types of hypoglycemic drugs among the four groups was conducted using an R×C contingency table.

#### 2.5.1 Structural magnetic resonance data statistics

Analysis of covariance (ANCOVA) was used to compare the differences among the four groups in hippocampal subfield volume while controlling for important covariates such as age, sex, years of education, and eTIV. Furthermore, two‐way ANOVA and post hoc analysis were used to determine the MCI × IR interaction effect and the main effects of MCI and IR among the four groups.

#### 2.5.2 Functional magnetic resonance data statistics

The rs‐fMRI neuroimaging statistical analyses were conducted using the DPABISurf software. The *z*‐transformed SBFC map for each hippocampal subfields with significant group differences was analyzed with one‐way ANOVA to compare the intergroup differences among the four groups. At the same time, age, gender, years of education, and FD values were used as covariates in this study. A total of 5000 nonparametric permutations were performed for each analysis, and threshold‐free cluster enhancement (TFCE) was applied. Multiple comparisons were corrected with family‐wise error rates (FWE) using a threshold of *p* < 0.025. FC values were extracted from regions of interest defined by the abnormal brain regions for further intergroup analysis. ANOVA and post hoc (Bonferroni‐corrected, *p* < 0.025) tests were applied to confirm the intergroup effect in the SPSS 20.0 software.

#### 2.5.3 Partial correlation and regression analyses

Partial correlation analyses were performed after including age, sex, and education as covariates, and eTIV was additionally included as a covariate in the analyses of hippocampal subfield volume, and FD was additionally included as a covariate in the analyses of FC values. Finally, a stepwise multiple linear regression analysis was also conducted to verify the correlation results.

## 3 RESULTS

### 3.1 Demographic data and neuropsychological tests

**FIGURE 2:**
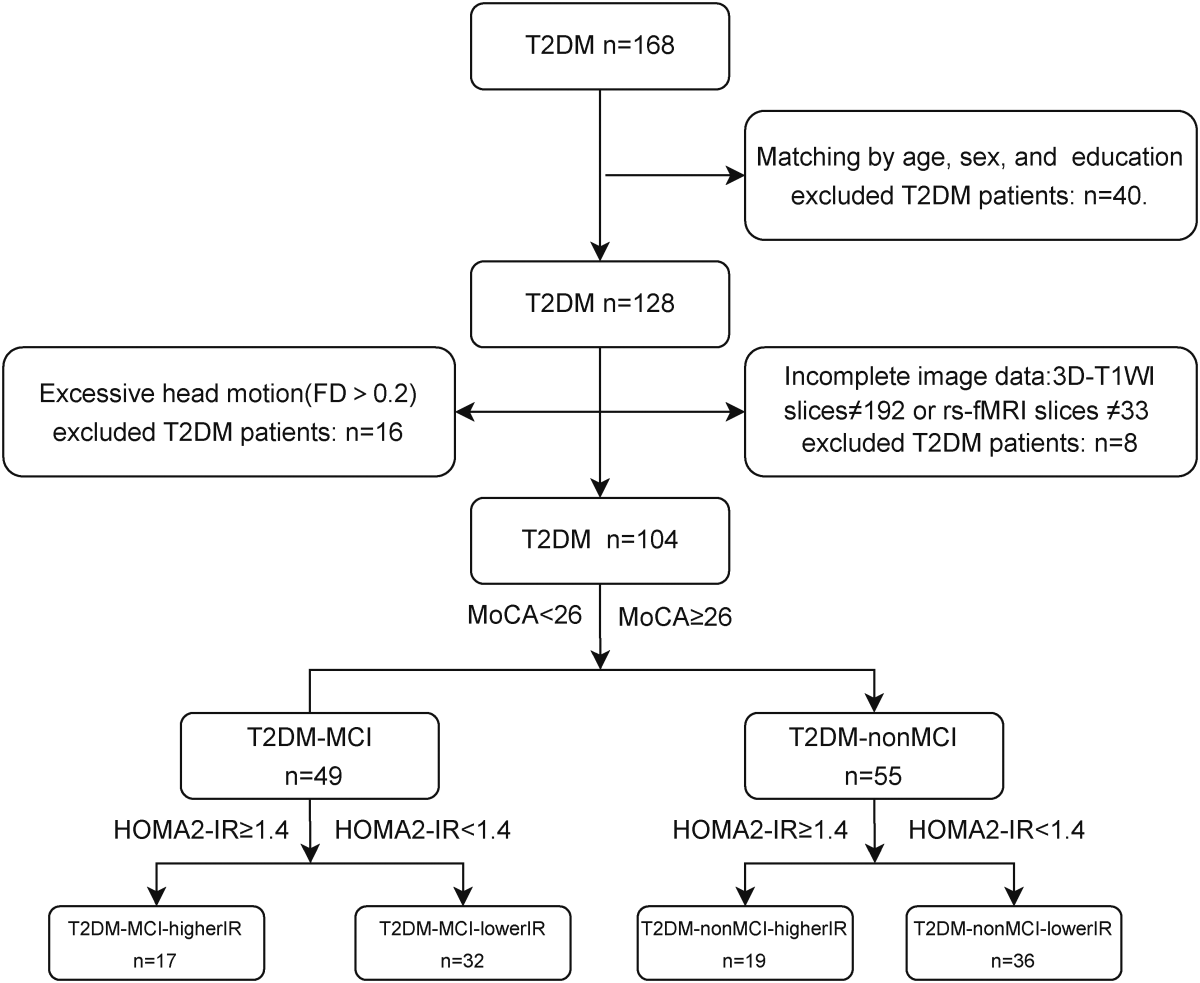
A flowchart shows participant selection. Matching by age, sex, and education excluded T2DM patients: five from the T2DM‐MCI‐higherIR group, eight from the T2DM‐MCI‐lowerIR group, 10 from the T2DM‐nonMCI‐higherIR group, and 17 from the T2DM‐nonMCI‐lowerIR group. Incomplete image data: two from the T2DM‐MCI‐higherIR group, three from the T2DM‐MCI‐lowerIR group, one from the T2DM‐nonMCI‐higherIR group, and two from the T2DM‐nonMCI‐lowerIR group. Excessive head motion: two from the T2DM‐MCI‐higherIR group, six from the T2DM‐MCI‐lowerIR group, three from the T2DM‐nonMCI‐higherIR group, and five from the T2DM‐nonMCI‐lowerIR group. FD, frame displacement; IR, insulin resistance; MCI, mild cognitive impairment; MoCA, Montreal Cognitive Assessment (Beijing vision); rs‐fMRI, resting state functional magnetic resonance imaging; T2DM, type 2 diabetes mellitus.

In this experiment, a total of 168 T2DM patients were collected. To minimize the effect of intergroup differences in age, sex, and education among the four groups in the preliminary stage, because the T2DM‐MCI‐higherIR group had fewer years of education, there were no statistical differences in sex, age, and education among the four groups after excluding 40 subjects. During data processing, eight T2DM patients were excluded from the study due to missing or incomplete data in 3D‐T1WI images or rs‐fMRI images. Additionally, 16 patients were excluded due to excessive head movement (FD >0.2). In total, 104 T2DM patients were included in the study (Figure 2).

Table 1 summarizes the differences between the demographic data and neuropsychological tests scores between the four groups, with statistical differences between the four groups for FINS, HOAM2‐IR, MoCA, and MMSE. Specifically, T2DM patients with IR had higher FINS and HOMA2‐IR (*p* < 0.001), and T2DM patients with MCI had lower MoCA and MMSE scores (*p* < 0.001). No differences were found in other clinical data and neuropsychological test results. Additionally, among the T2DM patients, only one subject in the T2DM‐MCI‐higherIR group, four subjects in the T2DM‐MCI‐lowerIR group, and one subject in the T2DM‐nonMCI‐lowerIR group had not taken any hypoglycemic agents. A significant difference in insulin analog use was observed among the four groups, without a significant difference in the use of other hypoglycemic medications (Supplementary Information 2).

**TABLE 1:** Demographic data, clinical characteristics, and neuropsychological test scores.

	T2DM‐MCI‐higher IR	T2DM‐MCI‐lower IR	T2DM‐nonMCI‐higher IR	T2DM‐nonMCI‐lower IR	*F*/*χ* ^2^/*H*	*p* value
	17	32	19	36		
Age (years)	54.71 ± 10.74	54.45 ± 7.17	54.10 ± 7.37	53.41 ± 7.55	0.147	0.932
Sex (male/female)	12/5	25/8	17/3	31/6	1.181	0.645
Education (years)	12 (9, 15)	12 (10.5, 15)	15 (12, 15)	15 (12, 15)	6.647	0.084
Duration (years)	10 (2.5, 15)	7 (5, 10.5)	10 (6.5,14.25)	7 (4.5, 10)	2.877	0.411
BMI	24.23 ± 2.98	23.96 ± 2.72	24.23 ± 2.74	24.87 ± 2.71	0.665	0.575
SBP (mmHg)	132.76 ± 23.41	126.61 ± 15.40	125.95 ± 16.32	127.78 ± 14.79	0.626	0.600
DPB (mmHg)	84.94 ± 10.85	78.88 ± 9.12	80.60 ± 8.40	85.16 ± 11.25	2.846	0.051
HbA1c (%)	8.82 ± 2.56	8.58 ± 2.38	9.22 ± 1.67	8.34 ± 1.93	0.780	0.508
FBG (mmol/L)	9.73 ± 5.01	10.02 ± 3.45	11.85 ± 3.17	9.45 ± 2.91	2.118	0.103
FINS (pmol/L)	12.20 (10.65, 15.70)	5.80 (3.95, 6.9)	12.90 (10.70, 18.20)	5.40 (4.25, 7.85)	55.245	<0.001
HOMA2‐IR	1.74 (1.60, 2.50)	0.85 (0.59, 1.10)	2.05 (1.88, 2.78)	0.78 (0.64, 1.19)	72.317	<0.001
CRP (mg/L)	1.60 (0.6, 5, 2.6)	1.00 (0.55, 1.90)	1.10 (0.53, 1.60)	1.00 (0.55, 2.05)	0.756	0.860
TC (mmol/L)	4.19 ± 0.83	4.16 ± 0.54	4.28 ± 0.77	4.57 ± 1.06	1.696	0.173
TG (mmol/L)	1.64 (1.37, 2.48)	1.47 (1.04, 2.03)	1.82 (1.15, 2.66)	1.78 (1.29, 2.18)	1.989	0.575
HDL (mmol/L)	1.00 ± 0.20	1.09 ± 0.24	1.02 ± 0.23	1.11 ± 0.28	1.157	0.330
LDL (mmol/L)	2.33 ± 0.49	2.33 ± 0.49	2.60 ± 0.89	2.61 ± 0.78	1.492	0.220
HAMD‐24 (scores)	8 (2.5, 14)	9 (4,13.5)	5.5 (2, 9)	6 (2, 8.5)	3.942	0.268
HAMA (scores)	5 (2.5, 8)	3 (1, 8.5)	2.5 (0, 6.25)	3 (1, 0.5)	4.370	0.224
MMSE (scores)	28.00 ± 1.41	27.27 ± 2.30	28.90 ± 1.17	28.94 ± 1.84	5.765	0.001
MoCA (scores)	24 (22, 24)	23 (23, 24.5)	28 (27, 29)	27 (26, 28)	80.816	<0.001

Abbreviations: BMI, body mass index; CRP, C‐reactive protein; DBP, diastolic blood pressure; FBG, fasting blood glucose; FINS, fasting insulin; HAMA, Hamilton Anxiety Scale; HAMD‐24, 24‐item Hamilton Depression Scale; HbA1c, hemoglobin A1c; HDL, high‐density lipoprotein; HOMA2‐IR, homeostasis model assessment insulin resistance; IR, insulin resistance; LDL, low‐density lipoprotein; MCI, mild cognitive impairment; MMSE, Mini‐mental State Examination; MoCA, Montreal Cognitive Assessment (Beijing version); SBP, systolic blood pressure; T2DM, type 2 diabetes mellitus; TC, total cholesterol; TG, triglyceride.

### 3.2 Volume of hippocampal subfields

**FIGURE 3:**
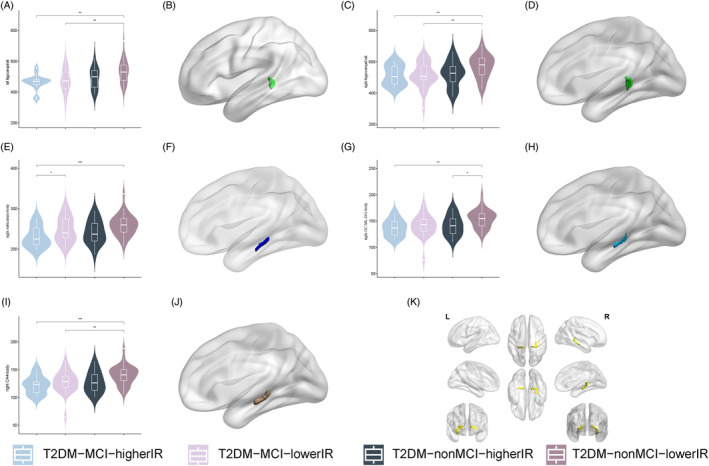
Comparison of hippocampal subfields volume among four groups. (A) Location of left hippocampal tail. (B) Intergroup differences in left hippocampal tail. (C) Location of right hippocampal tail. (D) Intergroup differences in right hippocampal tail. (E) Location of right subiculum‐body. (F) Intergroup differences in right subiculum‐body. (G) Location of right GC‐ML‐DG‐body. (H) Intergroup differences in right GC‐ML‐DG‐body. (I) Location of right CA4‐body. (J) Intergroup differences in right CA4‐body. (K) location of bilateral hippocampal tail, right subiculum‐body, right GC‐ML‐DG‐body, and right CA4‐body. CA, cornu ammonis; GC‐ML‐DG, granule cell layer of dentate gyrus; IR, insulin resistance; MCI, mild cognitive impairment; T2DM, type 2 diabetes mellitus.*p < 0.05; **p < 0.01; ***p < 0.001, post hoc: Bonferroni (*p* < 0.05).

Analysis of covariance revealed that left hippocampal tail (*p* = 0.006; Figure 3A,B), right hippocampal tail (*p* = 0.032; Figure 3C,D), right subiculum‐body (*p* = 0.024; Figure 3E,F), right GC‐ML‐DG‐body (*p* = 0.016; Figure 3G,H), and right CA4‐body (*p* = 0.014; Figure 3I,J) with between‐group differences (Supplemental Information 3) and post hoc tests showed that compared with the T2DM‐nonMCI‐lowerIR group, all five hippocampal subfields had lower volume in T2DM‐MCI‐higherIR group. In addition, hippocampal volumes were found to be smaller in the T2DM‐MCI‐lowerIR group than in the T2DM‐nonMCI‐lowerIR group in the bilateral tail and right CA4‐body, and in the right GC‐ML‐DG‐body, the T2DM‐nonMCI‐higherIR group was found to be smaller than the T2DM‐nonMCI‐lowerIR group. Moreover, the T2DM‐MCI‐higherIR group was found to be smaller than the T2DM‐MCI‐lowerIR group in the right subiculum‐body.

The two‐way ANOVA analysis detected no interaction effect among the groups, and a main effect of MCI in all five hippocampal subregion volumes with differences (*p* < 0.05), IR in right hippocampal tail (*p* = 0.003), right subiculum‐body (*p* = 0.025), and right GC‐ML‐DG‐body (*p* = 0.015; Table 2).

**TABLE 2:** Result of two‐way ANOVA.

	Left hippocampal tail	Right hippocampal tail	Right subiculum‐body	Right GC‐ML‐DG‐body	Right CA4‐body
*F*	8.517^a^/3.944^b^/1.224^c^	4.221^a^/2.871^b^/2.194^c^	4.362^a^/9.267^b^/0.011^c^	5.374^a^/5.182^b^/1.203^c^	5.659^a^/6.097^b^/0.895^c^
*P*	0.004^a^/0.050^b^/0.258^c^	0.043^a^/0.093^b^/0.142^c^	0.039^a^/0.003^b^/0.919^c^	0.022^a^/0.025^b^/0.275^c^	0.019^a^/0.015^b^/0.057^c^

Abbreviations: ANOVA, analysis of variance; GC‐ML‐DG, granule cell layer of dentate gyrus; IR, insulin resistance; MCI, mild cognitive impairment.

^a^ The main effect of mild cognitive impairment.

^b^ The main effect of insulin resistance.

^c^ The interaction effect of MCI × IR interaction effect.

### 3.3 Surface‐based functional connectivity

**FIGURE 4:**
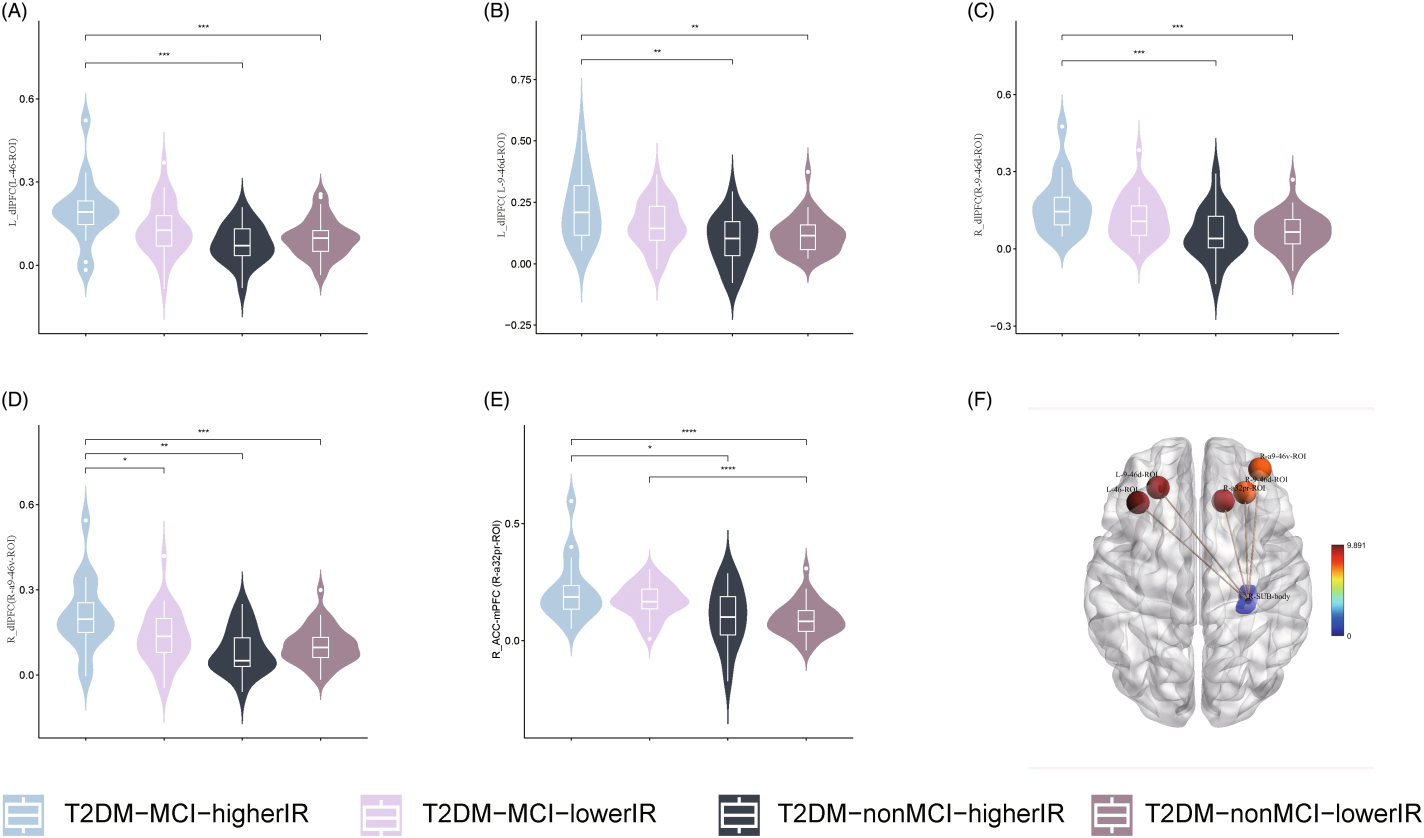
Comparison of FC value among four groups. (A) Intergroup differences in FC value between ROI and left dorsolateral prefrontal cortex (L‐46‐ROI). (B) Intergroup differences in FC value between ROI and left dorsolateral prefrontal cortex (L‐9‐46d‐ROI). (C) Intergroup differences in FC value between ROI and right dorsolateral prefrontal (R‐9‐46d‐ROI). (D) Intergroup differences in FC value between ROI and right dorsolateral prefrontal (R‐a9‐46v‐ROI). (E) Intergroup differences in FC value between ROI and right anterior cingulate–medial prefrontal cortex (R‐a32pr‐ROI). (E) Location of ROI and L‐46‐ROI, L‐9‐46d‐ROI, R‐9‐46d‐ROI, R‐a9‐46v‐ROI, and R‐a32pr‐ROI. FC, functional connectivity; IR, insulin resistance; MCI, mild cognitive impairment; ROI, region of interest; T2DM, type 2 diabetes mellitus. *p < 0.05; **p < 0.01; ***p < 0.001; ****p < 0.0001; TFCE (5000 permutations, FWE *p* < 0.025); post hoc: Bonferroni (*p* < 0.025).

The results of the SBFC analysis are depicted in Figure 4. In the left hemisphere, the FC was enhanced between the right subiculum‐body and the left dorsolateral prefrontal cortex (L‐46‐ROI/L‐9‐46d‐ROI; Figure 4A,B; Table 3). Our post hoc analysis revealed statistically significant differences among the T2DM‐MCI‐higherIR group, T2DM‐nonMCI‐higherIR group, and T2DM‐nonMCI‐lowerIR group (*p* < 0.05). In the right hemisphere, the FC was increased between the right dorsolateral prefrontal (R‐9‐46d‐ROI/R‐a9‐46v‐ROI; Figure 4C,D) and anterior cingulate–medial prefrontal cortex (R‐a32pr‐ROI; Figure 4E; Table 3). The post hoc analysis revealed differences among the T2DM‐MCI‐higherIR group, T2DM‐nonMCI‐higherIR group, and T2DM‐nonMCI‐lowerIR group (*p* < 0.05). A difference was also detected in the right dorsolateral prefrontal cortex (R‐a9‐46v‐ROI) between the T2DM‐MCI‐higherIR and T2DM‐MCI‐lowerIR groups (*p* < 0.05). In the anterior cingulate–medial prefrontal cortex (R‐a32pr‐ROI), a difference was observed between T2DM‐MCI‐lowerIR group and T2DM‐nonMCI‐lowerIR group. No statistically significant differences were found between the other ROIs and other brain regions in the whole brain.

**TABLE 3:** Abnormal FC in significant clusters among four groups.

Brain regions	HCP	Cluster size (mm^2^)	Peak MNI coordinate			Peak *F*‐value
			*x*	*y*	*z*	
Left dorsolateral prefrontal cortex (L_46_ROI)	84	138.279	−35.908	29.799	33.873	9.891
Left dorsolateral prefrontal cortex (L_9‐46d_ROI)	86	145.854	−23.766	38.702	27.772	9.573
Right dorsolateral prefrontal cortex (R_a9‐46v_ROI)	85	108.245	37.464	49.879	16.951	7.738
Right dorsolateral prefrontal cortex (R_9‐46d_ROI)	86	160.545	27.127	35.724	26.582	7.824
Right anterior cingulate–medial prefrontal cortex (R_a32pr_ROI)	179	112.705	14.724	31.267	25.150	9.620

Abbreviations: FC, functional connectivity; HCP, Human Connectome Project; MNI, Montreal Neurological Institute (statistics method: TFCE 5000 permutations, FWE‐correction *p* < 0.05).

### 3.4 Correlation analysis

**FIGURE 5:**
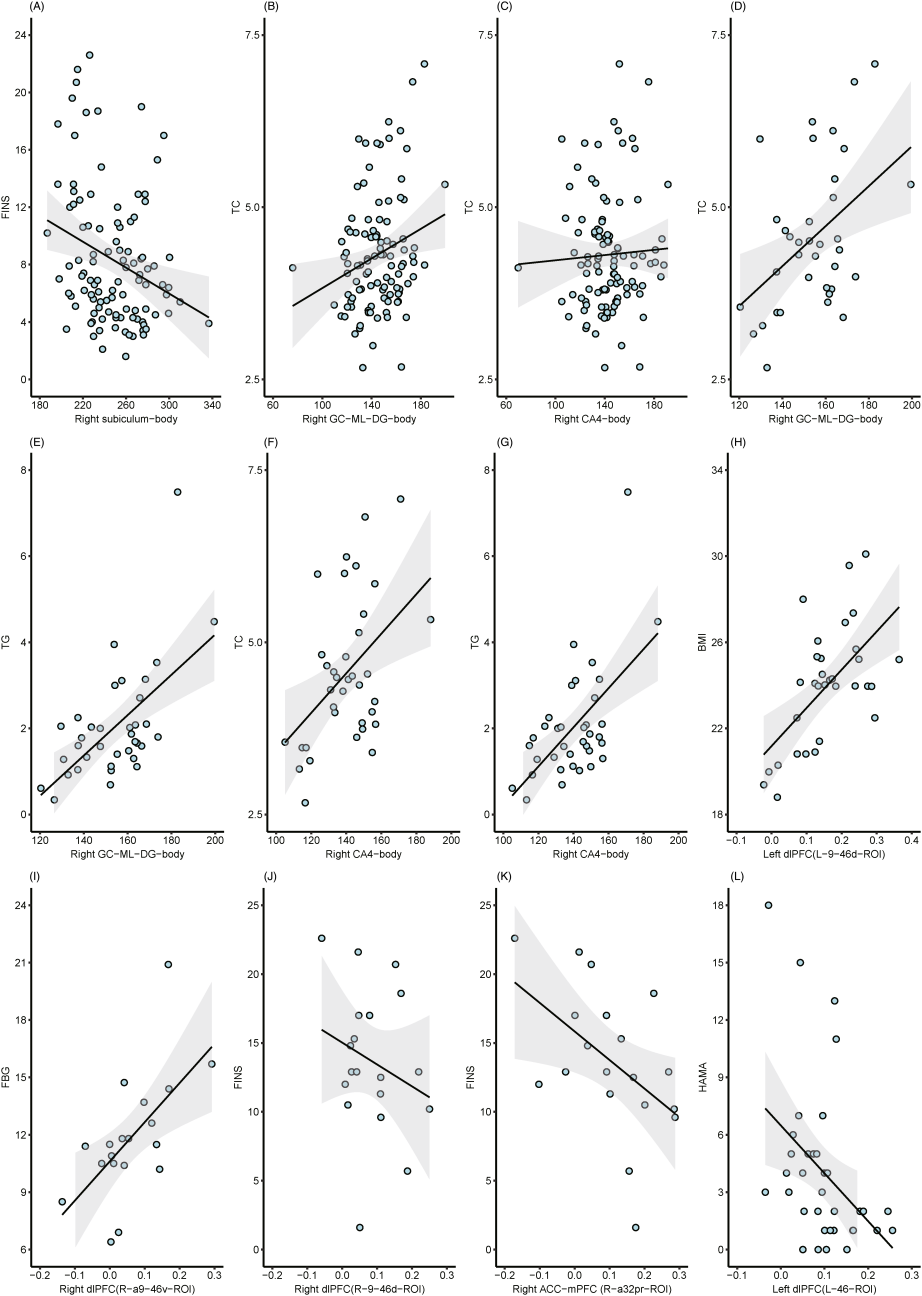
Correlation between demographic data and neuropsychological tests scores and neuroimaging results. (A) Correlation between right subiculum‐body and FINS in all T2DM patients (*r* = −0.297, *P*_*FDR*_ = 0.015). (B) Correlation between right GC‐ML‐DG‐body and TC in all T2DM patients (*r* = 0.377, *P*_*FDR*_ = 0.005). (C) Correlation between right CA4‐body and TC in all T2DM patients (*r* = 0.290, *P*_*FDR*_ = 0.010). (D) Correlation between right GC‐ML‐DG‐body and TC in T2DM‐nonMCI‐lowerIR group (*r* = 0.539, *P*_*FDR*_ = 0.009). (E) Correlation between right GC‐ML‐DG‐body and TG in T2DM‐nonMCI‐lowerIR group (*r* = 0.588, *P*_*FDR*_ = 0.006). (F) Correlation between CA4‐body and TC in T2DM‐nonMCI‐lowerIR group (*r* = 0.522, *P*_*FDR*_ = 0.018). (G) Correlation between CA4‐body and TG in T2DM‐nonMCI‐lowerIR group (*r* = 0.594, *P*_*FDR*_ = 0.005). (H) Correlation between right subiculum‐body‐left dorsolateral prefrontal cortex (L‐9‐46d‐ROI) values and BMI in T2DM‐MCI‐lowerIR group (*r* = 0.467, *P*_*FDR*_ = 0.03). (I) Correlation between right subiculum‐body‐right dorsolateral prefrontal cortex (R‐9‐46d‐ROI) FC values and FBG in T2DM‐MCI‐lowerIR group (*r* = 0.626, *P*_*FDR*_ = 0.03). (J) Correlation between right subiculum‐body‐right dorsolateral prefrontal cortex (R‐a9‐46v‐ROI) FC values and FINS in T2DM‐MCI‐lowerIR group (*r* = −0.553, *P*_*FDR*_ = 0.04). (K) Correlation between right subiculum‐body‐right anterior cingulate–medial prefrontal cortex (R‐a32pr‐ROI) and FINS in T2DM‐MCI‐lowerIR group (*r* = −0.557, *P*_*FDR*_ = 0.04). (L) Correlation between right subiculum‐body‐left dorsolateral prefrontal cortex (L‐46‐ROI) and HAMA in T2DM‐nonMCI‐lowerIR group (*r* = −0.465, *P*_*FDR*_ = 0.03). BMI, body mass index; FBG, fasting blood glucose; FC, functional connectivity; FINS, fasting insulin; HAMA, Hamilton Anxiety Scale; IR, insulin resistance; MCI, mild cognitive impairment; ROI, region of interest; TC, total cholesterol; TG, triglyceride; T2DM, type 2 diabetes mellitus.

Correlation analysis (Figure 4) revealed that after correction for false discovery rate (FDR), in all T2DM patients, a negative correlation existed between right subiculum‐body and FINS (*r* = −0.297, *P*_*FDR*_ = 0.015; Figure 5A) as well as right GC‐ML‐DG‐body and TC (*r* = 0.377, *P*_*FDR*_ = 0.005; Figure 5B), whereas a positive correlation existed between right CA4‐body and TC (*r* = 0.290, *P*_*FDR*_ = 0.010; Figure 5C). In the T2DM‐nonMCI‐lowerIR group, positive correlations were noted between right GC‐ML‐DG‐body and TC (*r* = 0.539, *P*_*FDR*_ = 0.009; Figure 5D), right GC‐ML‐DG‐body and TG (*r* = 0.588, *P*_*FDR*_ = 0.006; Figure 5E), right CA4‐body and TC (*r* = 0.522, *P*_*FDR*_ = 0.018; Figure 5F), and right CA4‐body and TG (*r* = 0.594, *P*_*FDR*_ = 0.005; Figure 5G). Correlation analysis of right subiculum‐body FC values with clinical variables identified positive correlations between left dorsolateral prefrontal cortex (L‐9‐46d‐ROI) and BMI (*r* = 0.467, *P*_*FDR*_ = 0.03; Figure 5H) in the T2DM‐MCI‐lowerIR group and between right dorsolateral prefrontal cortex (R‐9‐46d‐ROI) and FBG (*r* = 0.626, *P*_*FDR*_ = 0.03; Figure 5I) in the T2DM‐nonMCI‐higherIR group. On the other hand, a negative correlation was observed between right dorsolateral prefrontal cortex (R‐a9‐46v‐ROI) and FINS (*r* = −0.553, *P*_*FDR*_ = 0.04; Figure 5J) and anterior cingulate–medial prefrontal cortex (R‐a32pr‐ROI) and FINS (*r* = −0.557, *P*_*FDR*_ = 0.04; Figure 5K) in the T2DM‐nonMCI‐higherIR group, and left dorsolateral prefrontal cortex (L‐46‐ROI) and HAMA (*r* = −0.465, *P*_*FDR*_ = 0.03; Figure 5L) in the T2DM‐nonMCI‐lowerIR group.

### 3.5 Multiple linear regression

After multiple linear regression of the correlation results corrected for FDR, we found that in all included T2DM patients, FINS exerted a negative effect on the volume of the hippocampal subfield (*β* = −0.252, *p* = 0.003), while TC exerted a positive effect (*β* = 0.296, *p* = 0.001). In the T2DM‐nonMCI‐lowerIR group, both TC and TG had a positive impact on subfield volume changes (TC *β* = 0.559, *p* = 0.001; TG *β* = 0.599, *p* < 0.001; TC *β* = 0.563, *p* = 0.001; TG *β* = 0.588, *p* < 0.001). Additionally, in FC‐related regression analysis, we observed that BMI (*β* = 0.563, *p* = 0.001) and FBG (*β* = 0.571, *p* = 0.028) served as positive factors in FC changes in the T2DM‐MCI‐lowerIR group and in the T2DM‐nonMCI‐higherIR group, respectively, while FC alteration was a negative factor in HAMA scores (*β* = −0.437, *p* = 0.008) in the T2DM‐nonMCI‐lowerIR group.

## 4 DISCUSSION

Few previous studies have focused on the role of IR in the structural or functional impairments of hippocampal subfields in patients with T2DM‐MCI. In the present study, participants in the T2DM‐MCI‐higherIR group exhibited more severe atrophy in specific hippocampal subfields than those in other groups. Moreover, principal factor analysis demonstrated that IR as an independent factor significantly affected the volume changes of right hippocampal tail, right subiculum‐body, and right GC‐ML‐DG‐body. Additionally, T2DM‐nonMCI‐higherIR patients exhibited decreased FC in the dorsal prefrontal lobe and anterior cingulate–medial prefrontal cortex. Furthermore, these above structural and functional alterations were related to glucose and lipid metabolism indicators and anxious scores.

In present study, we found that participants in the T2DM‐MCI‐higherIR group had the smallest hippocampal subfields volume among the four groups, and the principal factor analysis indicated that IR negatively impacts changes in the volume of the hippocampal subfield. Previous studies have shown that IR promotes Aβ protein aggregation and tau phosphorylation,^43^ further disrupting insulin function and exacerbating AD pathology. Furthermore, the role of IR as a risk factor for brain impairment of neurodegenerative disease is now well established,^44^ Because both AD and T2DM patients have brain damage closely related to IR, AD has been termed as “type 3 diabetes mellitus”,^44^ The use of insulin sensitizers to improve IR can significantly improve cognitive performance of AD patients.^45^, ^46^ Therefore, the results of the present study further expand the understanding of IR in the alteration of hippocampal subfields in T2DM patients, as well as complementing previous studies. Our findings suggest that hippocampal structural and functional damage in T2DM‐MCI patients may be associated with IR. This provides a potential therapeutic target for improving hippocampal impairment in T2DM patients, and offers a mechanistic basis for improving the long‐term prognosis of T2DM patients. However, the assessment of the HOMA2‐IR index may be affected by the use of insulin analogues and insulin sensitizers, which needs to be investigated in further studies.

Tri‐synaptic circuitry have an important role in information processing in the hippocampus, in which DG granular neurons connect via mossy fibers with CA3 pyramidal neurons that project via Schaffer collaterals to CA1 and to subiculum.^47^, ^48^ Our experiments revealed that the volume of right subiculum body and GC‐DG‐ML was smaller in T2DM‐MCI‐higherIR than T2DM‐nonMCI‐lowerIR groups. As an key component of synaptic structures, the subiculum is responsible for the primary output of the hippocampus to other areas, while the DG receives information from the entorhinal cortex. We speculate that damage to hippocampal information inflow and outflow pathways may relate to cognitive impairment.^31^, ^49^ Previous studies have also demonstrated that atrophy of the subiculum is considered to be the earliest hippocampal anatomical marker in AD.^50^ Thus, subiculum and GC‐DG‐ML also have the potential to be a biological marker for predicting brain impairment in patients with T2DM.

Another crucial finding of this study is increased FC between the subiculum and bilateral dorsolateral prefrontal, right anterior cingulate‐middle prefrontal gyrus in the T2DM‐MCI‐higherIR group. Considerable evidence suggests that a bidirectional flow of information is reflected by oscillatory synchrony between the prefrontal cortex and hippocampus, which is called the hippocampal–prefrontal circuitry.^51^, ^52^ It has been proposed to be involved in learning and memory. In addition, the anterior cingulate–middle prefrontal gyrus has a high degree of cooperativity, and they harmonize cognitive and emotional functions.^53^ Taken together, we speculate that the increase in FC may be a compensatory mechanism for the maintenance of cognitive function in T2DM patients.

The hippocampus is one of the adult brain regions with the capacity for neurogenesis, and the dentate gyrus granule cell layer has the capacity for continuous generation.^54^, ^55^ We observed a reduction in the GC‐DG‐ML in the right hippocampus. It has also been shown that neurogenesis in the hippocampal dentate gyrus is reduced in DM mice,^56^ which further substantiated the present results. Insulin promotes neurogenesis and regulates synaptic plasticity in the central nervous system. Overall, a decrease in insulin action due to IR would affect the ability of the dentate gyrus granule cell layer to generate, which may be a potential neuropathological mechanism for the reduction in GC‐ML‐DG volume in T2DM‐nonMCI‐higherIR patients. Moreover, rodent experiments have demonstrated that reduced dentate gyrus volume is associated with poorer discriminatory memory capacity,^57^ which further suggests that reduced GC‐ML‐DG volume might be an important factor in the cognitive decline of T2DM patients.

Our results have demonstrated smaller volumes in CA4 and bilateral hippocampal tails in T2DM‐MCI‐higherIR than in T2DM‐nonMCI‐lowerIR group. These changes were also found in previous studies of the hippocampus in patients with major depressive disorder,^58^ possibly associated with occlusion of the penetrating arteries.^59^, ^60^ Besides brain IR, the burden of cerebral small vessel disease in T2DM patients has been shown in previous studies.^61^ Abnormalities in perfusion are often found in T2DM due to the effects of chronic hyperglycemia and complications. In the caudal region, the terminal segment of small arteries is particularly susceptible to perfusion impairment due to hypoxia as a result of the effects of severe folding of the CA1 zone and stress vasoconstriction.^62^ Regrettably, the difference in the volume of CA1 did not reach statistical significance in our study, and thus, further study is warranted.

The present study found that structural and functional alterations in hippocampal subfields positively correlated with TC, TG, BMI, and FBG and negatively correlated with FINS. Moreover, functional alteration was correlated with anxiety symptoms. Previous literature had reported that IR can act by several pathways to perturb lipid metabolism,^63^, ^64^ However, our finding was different from the above study, as our results indicated that the volume and FC of specific hippocampus subfields were positively correlated with TG and TC. This is probably because cholesterol is the most abundant component of myelin, which is essential for the assembly of myelin membranes in the central nervous system.^65^ An increase in cholesterol levels in the normal range is beneficial to the development of the nervous system. It is important to note that the mean cholesterol in our group was in the normal range.^66^ Within the normal ranges, it will exert a positive influence on T2DM patients. We also found that higher FBG, lower FINS (which means severer IR), and higher anxiety score were positively correlated with hippocampal subfield structural and functional alterations, suggesting that the associated neural compensatory mechanisms might be more active under the influence of a more disturbed glucose metabolism.

Our study has a few limitations that should be addressed. First, this study is a cross‐sectional study and does not allow for a deeper exploration of the longitudinal neurological changes and mechanisms involved. Second, due to the diversity of treatment regimens in T2DM patients, a high degree of heterogeneity exists in the treatment regimens of the patients included in our study. To overcome this shortcoming, we will conduct subgroup analyses for different drug regimens after expanding the sample size in order to investigate the effects of different treatment regimens on brain structure and function in T2DM patients. Particularly, the calculation of HOMA2‐IR may be affected by long‐acting insulin analogues and insulin sensitizers. In order to address this issue, we will increase the sample size and perform further subgroup analyses based on the use of different hypoglycemic agents (e.g., insulin analogs and insulin sensitizers) and collect longitudinal data to obtain more reliable results.

This study included single‐center Asian yellow population T2DM patients and obtained that T2DM‐MCI patients with higher IR have more severe structural and functional damage of hippocampal subfields. Although we used more stringent included criteria and more rigorous statistical methods to improve the reproducibility of the results of this study, however, due to the heterogeneity of T2DM patients across regions and ethnicities, in addition to individual differences in T2DM treatment plans, we still need to collect multicenter data with large samples to verify the stability of the present results.

## 5 CONCLUSION

In conclusion, T2DM‐MCI patients with IR had more severe atrophy and functional impairments in specific hippocampal subfields. IR may have an independent negative effect on the alterations in the hippocampal subfield of T2DM based on principal factor analysis. These findings support the importance of IR in T2DM‐MCI patients and underline the potential role of novel therapeutic strategies targeting IR.

## AUTHOR CONTRIBUTIONS

Chen Yang: conceptualization, writing—original draft, formal analysis, and visualization; Zihan Ma, Huiyan Zhang, and Jian Tan: methodology and software; Jing Tian, Jiancang Cao, Yannan Xu, and Yanjun Fan: investigation; Wenwen Zhang and Guang Huang: supervision and project administration; Lianping Zhao: writing—review and editing, conceptualization, resources, and funding acquisition.

## FUNDING INFORMATION

This work was supported by the National Natural Science Foundation of China (82360343), the “Young Scholars in Western China” project of the Chinese Academy of Sciences in 2020, the Natural Science Foundation of Gansu Province (20JR5RA156, 21JR7RA593), and the Research Fund of Gansu Provincial Hospital (22GSSYD‐76).

## CONFLICT OF INTEREST STATEMENT

The authors declare no conflicts of interest.

## Supporting information


**Data S1.** Supporting Information.

**Data S2.** Supporting Information.

**Data S3.** Supporting Information.
