# Supplementary material for: Structural and functional alterations of the hippocampal subfields in T2DM with mild cognitive impairment and insulin resistance: A prospective study
Source: J Diabetes. 2024 Nov 13;16(11):e70029. doi: 10.1111/1753-0407.70029 (PMC11560383; doi:10.1111/1753-0407.70029)
Supplement: Supplementary file 1 — Data S1. Supporting Information. [file JDB-16-e70029-s003.docx]

Functional MRI data preprocessing

Preprocessing of Whole-Brain MRI Data The anatomical and rs-fMRI data were preprocessed using DPABISurf V1.7, which is based on the Statistical Parametric Mapping software (SPM12, http://www.fil.ion.ucl.ac.uk/spm12) on the Matrix Laboratory platform (MATLAB R2018b, https:// www.mathworks.com/) software to automated surface-based cortical segmentation and subcortical volume-based segmentation. First, all MRI data were converted from DICOM format to NIfTI format, and the first 10 time points were discarded. Then, the data were converted into BIDS format.

Anatomical Data Calculation

The structural image processing consisted of the following steps: ⑴ the 3D-T1WI image was corrected for intensity nonuniformity and used as T1w-reference throughout the workflow, ⑵ the T1w-reference was then skull-stripped, (3) brain tissue segmentation of cerebrospinal fluid (CSF), white matter (WM), and gray matter was performed on the brain-extracted T1w , ⑷ brain surfaces were reconstructed using a recon-all command in FreeSurfer (version 6.0.1), ⑸ cortical surface-based spatial normalization from individual native space to fsaverage space (FreeSurfer reconstruction nomenclature), subcortical volume-based spatial normalization to Montreal Neurological Institute (MNI) standard space, and ⑹ the cortical thickness of the whole brain was calculated as a structural indicator.

Functional Data Calculation

The rs-fMRI reference was co-registered with the T1w-reference using bbregister (FreeSurfer), which implements boundary-based registration. The functional image processing steps were as follows: ⑴ a reference volume and its skull-stripped version were generated using a custom methodology of fMRIPrep, ⑵ slice timing was corrected using 3dTshift , ⑶ the rs-fMRI time series of cortex were resampled onto the following surfaces (FreeSurfer reconstruction nomenclature): fsaverage5 and the rs-fMRI time series of subcortex were resampled into standard space, generating a preprocessed rs-fMRI run in MNI space, ⑷ head motion correction, the mean framewise displacement (FD) per participant was obtained and when the mean FD was higher than 0.2 were excluded, ⑸ nuisance correction by regressing out the WM and CSF mean time series according to the WM and CSF masks segmented by FreeSurfer as well as the Friston-24 motion time series and global signal , ⑹ bandpass temporal filtering (0.01–0.1 Hz) and spatial smoothing (full-width at half-maximum of 6 mm) were applied to the functional images.

Table S1 Result of Two-way ANOVA

|  | Left hippocampal tail | Right hippocampal tail | Right subiculum-body | Right GC-ML-DG-body | Right CA4-body |
| --- | --- | --- | --- | --- | --- |
| F | 8.517^a^/3.944^b^/1.224^c^ | 4.221^a^/2.871^b^/2.194^c^ | 4.362^a^/9.267^b^/0.011^c^ | 5.374^a^/5.182^b^/1.203^c^ | 5.659^a^/6.097^b^/0.895^c^ |
| *P* | 0.004^a^/0.050^b^/0.258^c^ | 0.043^a^/0.093^b^/0.142^c^ | 0.039^a^/0.003^b^/0.919^c^ | 0.022^a^/0.025^b^/0.275^c^ | 0.019^a^/0.015^b^/0.057^c^ |

a, mild cognitive impairment; b, insulin resistance; c, MCI x IR interaction effect.

Table S2. Abnormal FC in significant clusters among four groups.

| Brain regions | HCP | Cluster size(mm^2^) | Peak MNI coordinate | | | Peak *F*-value |
| --- | --- | --- | --- | --- | --- | --- |
|  |  |  | x | y | z |  |
| left dorsolateral prefrontal cortex (L_46_ROI) | 84 | 138.279 | -35.908 | 29.799 | 33.873 | 9.891 |
| left dorsolateral prefrontal cortex  (L_9-46d_ROI) | 86 | 145.854 | -23.766 | 38.702 | 27.772 | 9.573 |
| right dorsolateral prefrontal cortex  (R_a9-46v_ROI) | 85 | 108.245 | 37.464 | 49.879 | 16.951 | 7.738 |
| right dorsolateral prefrontal cortex  (R_9-46d_ROI) | 86 | 160.545 | 27.127 | 35.724 | 26.582 | 7.824 |
| right anterior cingulate–medial prefrontal cortex  (R_a32pr_ROI) | 179 | 112.705 | 14.724 | 31.267 | 25.150 | 9.620 |

FC, functional connectivity; HCP, Human Connectome Project; MNI, Montreal Neurological Institute; TFCE 5000 permutations, FWE *P*<0.05
