# Supplementary material for: Structural and functional alterations of the hippocampal subfields in T2DM with mild cognitive impairment and insulin resistance: A prospective study
Source: J Diabetes. 2024 Nov 13;16(11):e70029. doi: 10.1111/1753-0407.70029 (PMC11560383; doi:10.1111/1753-0407.70029)
Supplement: Supplementary file 2 — Data S2. Supporting Information. [file JDB-16-e70029-s001.docx]

Supplementary Information 2

Table S2.The history of hypoglycemic agents in T2DM patients

|  | Metformin | Glucosidase inhibitor | Sulfonamides | Glinides | DPP4- inhibitors | SGLT-2 inhibitors | Insulin analogs | Traditional Chinese Medicine |
| --- | --- | --- | --- | --- | --- | --- | --- | --- |
| T2DM-MCI-higherIR group | 6/17 | 7/17 | 0/17 | 0/17 | 0/17 | 0/17 | 8/17 | 1/17 |
| T2DM-MCI-lowerIR group | 19/32 | 7/32 | 4/32 | 0/32 | 1/32 | 2/32 | 9/32 | 2/32 |
| T2DM-nonMCI-highIR group | 12/19 | 9/19 | 2/19 | 0/19 | 2/19 | 1/19 | 12/19 | 0/19 |
| T2DM-nonMCI-lowerIR group | 27/36 | 14/36 | 5/36 | 4/36 | 10/36 | 1/36 | 9/36 | 4/36 |
| χ^2^ Value | 7.788 | 4.179 | / | / | / | / | 9.661 | / |
| *P* value | 0.051 | 0.243 | / | / | / | / | 0.022* | / |

T2DM,Type 2 diabetes mellitus; MCI, Mild cognitive impairment; IR, Insulin resistance; *, The difference was statistically significant; /, the four groups could not been compared
