# Supplementary material for: Structural and functional alterations of the hippocampal subfields in T2DM with mild cognitive impairment and insulin resistance: A prospective study
Source: J Diabetes. 2024 Nov 13;16(11):e70029. doi: 10.1111/1753-0407.70029 (PMC11560383; doi:10.1111/1753-0407.70029)
Supplement: Supplementary file 3 — Data S3. Supporting Information. [file JDB-16-e70029-s002.docx]

Supplementary Information 3

Table S3.Volume comparison of differential hippocampal subfields of T2DM patients

|  | T2DM-MCI-higherIR group  (95% CI) | T2DM-MCI-lowerIR  Group  (95% CI) | T2DM-nonMCI-higherIR  Group  (95% CI) | T2DM-nonMCI-lowerIR  Group  (95% CI) | *F*-value | *P*-value |
| --- | --- | --- | --- | --- | --- | --- |
| Left hippocampal tail | 465.97±17.00  (432.23 to 499.70) | 476.72±12.81（452.73 to 500.71） | 491.75±15.78  (460.42 to 523.08) | 529.02±11.57  (506.06 to 551.98) | 4.447 | 0.006 |
| Right hippocampal tail | 515.83±17.77  (480.55 to 551.13) | 518.50±12.64  (493.41 to 543.59) | 526.49±16.51  (493.72 to 559.52) | 565.50±12.10  (541.40 to 589.72) | 3.064 | 0.032 |
| Right subiculum-body | 233.02±6.18  (220.76 to 245.77) | 249.34±4.39  (232.10 to 258.06) | 243.48±5.74  (232.09 to 254.87) | 255.81±4.20  (247.46 to 264.15) | 3.283 | 0.024 |
| Right GC-ML-DG-body | 138.11±4.04  (130.17 to 146.19) | 142.13±2.87  (136.42 to 147.82) | 142.70±3.75  (135.26 to 150.14) | 152.25±2.74  (146.79 to 157.70) | 3.600 | 0.016 |
| Right CA4-body | 123.43±4.11  (115.27 to 131.60) | 128.55±2.93  (122.74 to 134.35) | 128.63±3.82  (121.04 to 136.21) | 138.45±2.80  (132.90 to 144.01) | 3.716 | 0.014 |

T2DM, type 2 diabetes mellitus; MCI, mild cognitive impairment; IR, insulin resistance; CI, confidence interval; GC-ML-DG, granule cell layer of dentate gyrus.
